# Loss of p19^Arf^ Facilitates the Angiogenic Switch and Tumor Initiation in a Multi-Stage Cancer Model via p53-Dependent and Independent Mechanisms

**DOI:** 10.1371/journal.pone.0012454

**Published:** 2010-08-27

**Authors:** Danielle B. Ulanet, Douglas Hanahan

**Affiliations:** 1 Diabetes Center, University of California San Francisco, San Francisco, California, United States of America; 2 Helen Diller Family Comprehensive Cancer Center, University of California San Francisco, San Francisco, California, United States of America; 3 Department of Biochemistry and Biophysics, University of California San Francisco, San Francisco, California, United States of America; Dresden University of Technology, Germany

## Abstract

The Arf tumor suppressor acts as a sensor of oncogenic signals, countering aberrant proliferation in large part via activation of the p53 transcriptional program, though a number of p53-independent functions have been described. Mounting evidence suggests that, in addition to promoting tumorigenesis via disruptions in the homeostatic balance between cell proliferation and apoptosis of overt cancer cells, genetic alterations leading to tumor suppressor loss of function or oncogene gain of function can also incite tumor development via effects on the tumor microenvironment. In a transgenic mouse model of multi-stage pancreatic neuroendocrine carcinogenesis (PNET) driven by inhibition of the canonical p53 and Rb tumor suppressors with SV40 large T-antigen (Tag), stochastic progression to tumors is limited in part by a requirement for initiation of an angiogenic switch. Despite inhibition of p53 by Tag in this mouse PNET model, concomitant disruption of Arf via genetic knockout resulted in a significantly accelerated pathway to tumor formation that was surprisingly not driven by alterations in tumor cell proliferation or apoptosis, but rather via earlier activation of the angiogenic switch. In the setting of a constitutional p53 gene knockout, loss of Arf also accelerated tumor development, albeit to a lesser degree. These findings demonstrate that Arf loss of function can promote tumorigenesis via facilitating angiogenesis, at least in part, through p53-independent mechanisms.

## Introduction

The ARF (alternative reading frame; p14^ARF^ in human, p19^Arf^ in mouse) tumor suppressor serves as a sensor of hyper-proliferative signals, resulting in p53-dependent growth arrest and apoptosis [1], [2], [3]. While ARF is not expressed at appreciable levels in most normal tissues, oncogene activation triggers its expression [4], resulting in inhibition of the MDM2 ubiquitin ligase and stabilization of p53 [5]. Inhibition of the p53 pathway, most commonly via mutations in *p53* itself, inactivation of *ARF*, or amplification of *MDM2* is thought to be a critical step in the pathogenesis of most human cancers [6]. Although mutation of *p53* and *ARF* in tumors are for the most part mutually exclusive events [7], mounting evidence suggests that the relationship between p53 and ARF is not strictly linear and points to p53-independent tumor suppressor functions of ARF [8], [9]. Initial in vivo evidence for a potential disconnect between ARF and p53 came from the finding that combined *p19^Arf^* and *p53* deficiency in mice results in the emergence of a tumor spectrum consisting of a wider range of tumor types than in mice lacking either gene alone [9]. Subsequently, a study in the DMBA/TPA two-step mouse skin carcinogenesis model revealed an accelerated growth rate of papillomas in a *p19^Arf^*-deficient compared to a *p53*-deficient genetic background [10]. Similarly, *p19^Arf^* but not *p53* deficiency facilitated melanoma development in a transgenic mouse model through relief of a p53-independent senescence pathway [11]. A more recent study suggested a context-dependence to the outcome of *p19^Arf^* loss during oncogenesis in the setting of *Pten*-deficiency, in that *p19^Arf^* loss imparted a partial inhibitory function during prostate carcinogenesis, but abolished senescence and promoted hyperproliferation and transformation of mouse embryo fibroblasts. In both settings the differential outcome of *p19^Arf^* loss occurred without effects on p53 activity [12].

In a transgenic mouse model of multi-stage pancreatic neuroendocrine carcinogenesis (PNET), RIP-Tag2, expression of the SV-40 T-antigen (Tag) in pancreatic β cells leads to inhibition of the tumor suppressors p53 and Rb, promoting a temporally defined yet stochastic multi-stage pathway to tumorigenesis. Despite oncogene expression in all ∼400 mouse pancreatic islets, secondary events are required for progression to each step of the pathway to tumors [13], [14]. The majority, but not all normal islets develop into hyperplastic/dysplastic lesions throughout the approximate 14 week lifespan of RIP-Tag2 mice. Similarly, only a subset (∼15–20%) of hyperplastic islet lesions undergo an angiogenic switch, and only a fraction (∼25%) of these angiogenic islets subsequently progress into solid tumors. Promotion of the hyperplastic switch is thought to be in part mediated by the growth/survival factor, IGF-II, as its expression is induced concomitant with the onset of β-cell hyper-proliferation [15]. A more recent study has identified an association between microRNA expression signatures and the different RIP-Tag2 tumorigenesis stages, with the implication that alterations in the expression of these miRNAs could be playing a role in driving these “switches” [16].

We sought in this investigation to ascertain whether Arf had p53-independent functions in suppressing tumor progression in the RIP-Tag2 PNET model, via crosses to mice carrying a genetic knock-out of *Arf.* A previous study showed that genetic knockout of *p53* in this model did not result in an accelerated pathway to tumor formation, and in fact, beta tumor cell proliferation was reduced, likely a consequence of destabilization of the SV-40 large T antigen oncogene, to which it binds [17]. In contrast, we demonstrate here that loss of *Arf* significantly accelerates tumor formation, via facilitating the angiogenic switch. These studies provide *in vivo* evidence for p53-independent functions of Arf and suggest a novel role for Arf as a suppressor of tumor angiogenesis.

## Results

### Knockout of *Arf* accelerates RIP-Tag2 tumor progression without significant effects on tumor cell apoptosis/proliferation

**Figure 1:**
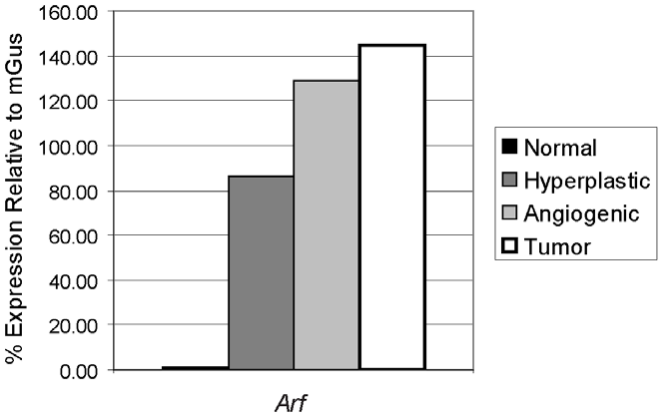
Expression of *Arf* mRNA during RIP-Tag2 tumor progression. The mRNA levels of *Arf* were assessed using quantitative RT-PCR on cDNA generated from RNA pools of normal non-transgenic islets, and hyperplastic islets, angiogenic islets (isolated islets from 5–10 mice were pooled), and tumors (equal amounts of RNA isolated from 10 tumors from at least 5 mice pooled) from RIP-Tag2 mice. Gene expression normalized and plotted relative to mGus expression.

**Figure 2:**
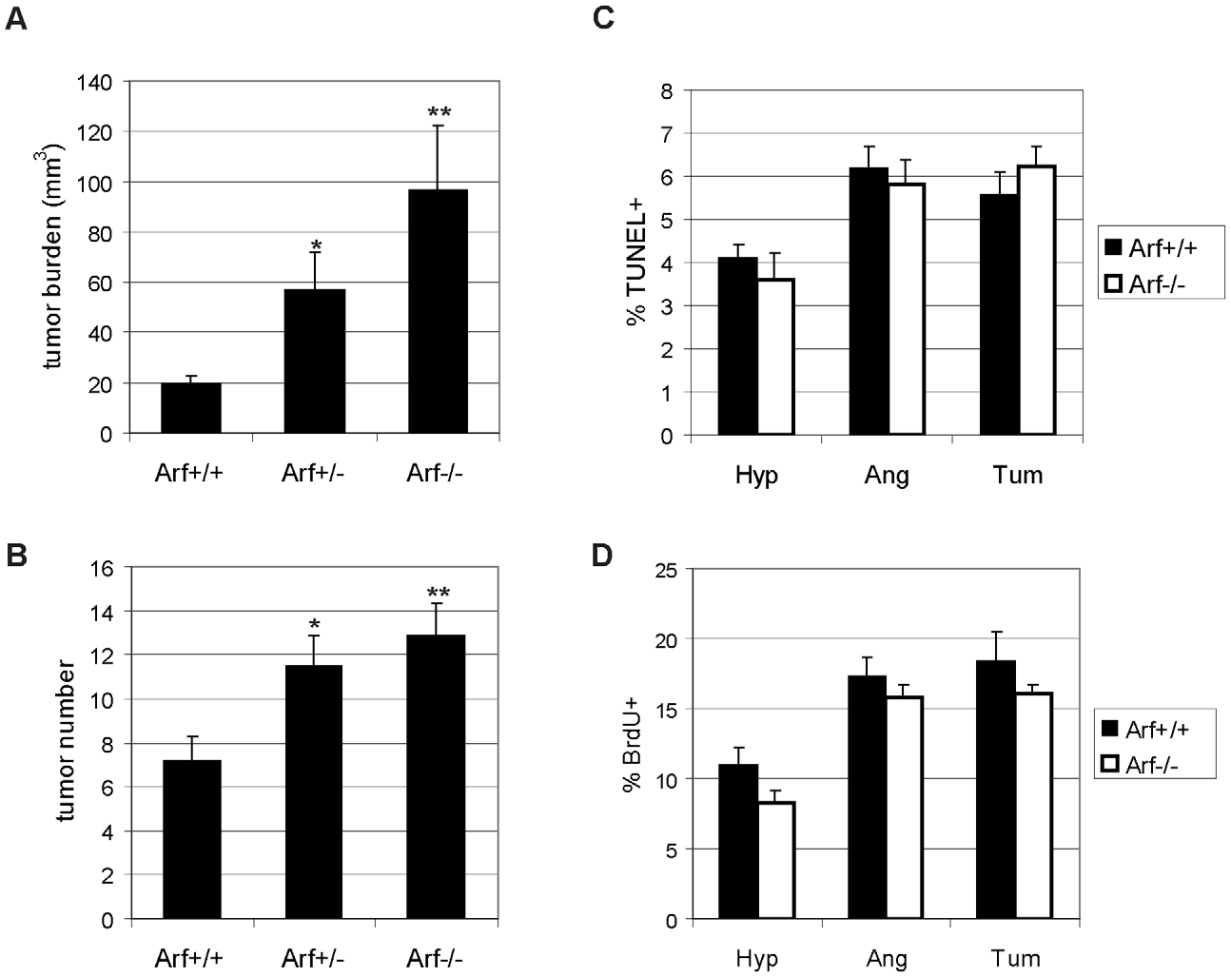
Genetic knockout of *Arf* accelerates RIP-Tag2 tumor progression without significantly affecting apoptosis/proliferation. (A,B) Effects of heterozygous and homozygous knockout of *Arf* on tumor burden (A; *p<0.001, **p<0.0001 compared to *Arf+/+*; the difference between *Arf+/−* and *Arf−/−* mice was not statistically significant) and tumor number (B; *p = 0.02, **p = 0.002) in 12 week-old RIP-Tag2 mice. Mean values +/− SEM from 15 (*Arf+/+*), 17 (*Arf+/−*), or 20 (*Arf−/−*) mice/group are indicated. Mann-Whitney test for statistical significance. (C) Apoptosis, as determined by TUNEL labeling in hyperplastic islets (Hyp), angiogenic islets (Ang), and tumors (Tum) from *Arf^+/+^* or *Arf^−/−^* RIP-Tag2 mice. Mean values +/− SEM from graded lesions of 6–7 individual mice of each genotype are indicated. The number of total lesions analyzed for each genotype/lesion stage are as follows: *Arf+/+* (Hyp: n = 27; Ang: n = 33; Tum: n = 14); *Arf−/−* (Hyp: n = 18; Ang: n = 25: Tum: n = 22). (D) Proliferation, as measured by the % of cells with BrdU incorporation in the different stages of islet lesions from 4 individual *Arf+/+* (Hyp: n = 21; Ang: n = 24; Tum: n = 12) and *Arf−/−* (Hyp: n = 18; Ang: n = 22; Tum: n = 24) RIP-Tag2 mice.

Consistent with the known induction of p19^Arf^ in response to oncogenic signals [4], *Arf* mRNA expression was found to be induced in SV-40 Tag expressing hyperproliferative islets compared to normal islets, with a further increase with progression to angiogenic lesions and solid tumors in RIP-Tag2 mice (Figure 1). To determine whether Arf might be suppressing tumorigenesis in the setting of p53 inhibition, we assessed the effects of genetic knockout of *Arf* on this pathway. Remarkably, loss of Arf resulted in a very significant, nearly 5-fold increase in tumor burden (Figure 2A, p<0.0001); there was, in addition, a 1.8-fold increase in tumor number (Figure 2B, p = 0.002). Heterozygous loss of Arf also resulted in a significant increase in tumor burden (p<0.001) and tumor number (p = 0.02) compared to *Arf* wild-type RIP-Tag2 mice. Loss of the wild-type *Arf* allele did not occur in any of the *Arf^+/−^* tumors examined (Figure S1).

**Figure 3:**
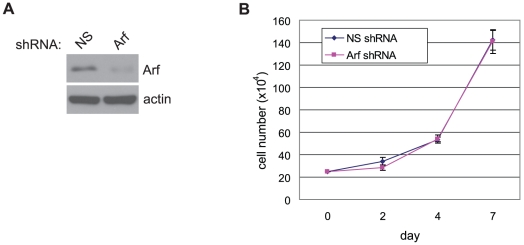
Knockdown of *Arf* does not affect growth of SV-40 Tag oncogene expressing β-tumor cells. Tumor cells were stably transduced with a non-silencing (NS) or *Arf* silencing shRNA. (A) Knockdown of Arf protein expression by the *Arf* targeting shRNA. β-actin protein levels indicated as a control for protein load. (B) Effect of *Arf* knockdown on tumor cell growth *in vitro*. Mean values +/−SEM from three independent experiments performed in triplicate are indicated.

Given the previously demonstrated ability of ARF to induce cell cycle arrest and apoptosis independently of p53 [9], [18], [19], [20], [21], we examined for changes in apoptosis and proliferation as assessed by TUNEL labeling and BrdU incorporation, respectively, in pre-neoplastic and tumor lesions from *RIP-Tag2; Arf^+/+^* and *Arf^−/−^* mice. Surprisingly, we were unable to detect any significant effect of loss of Arf on either of these parameters at any of the RIP-Tag2 stages (Figure 2C, D). To ensure that we did not overlook subtle effects on cell cycle regulation/proliferation, in addition to the BrdU incorporation assay, which measures the percentage of cells in S-phase, we also assessed the proliferative index by immunostaining for phospho-histone H3, a marker for cells in M-phase. In agreement with the BrdU-incorporation assay, no significant difference in the percentage of cells in M-phase was observed upon loss of Arf (Figure S2). Furthermore, stable knockdown of *Arf* using an *Arf* targeting shRNA in a cell line derived from a wild-type RIP-Tag2 tumor had no effect on growth of the cancer cells in vitro (Figure 3).

### Loss of Arf facilitates the angiogenic switch

**Figure 4:**
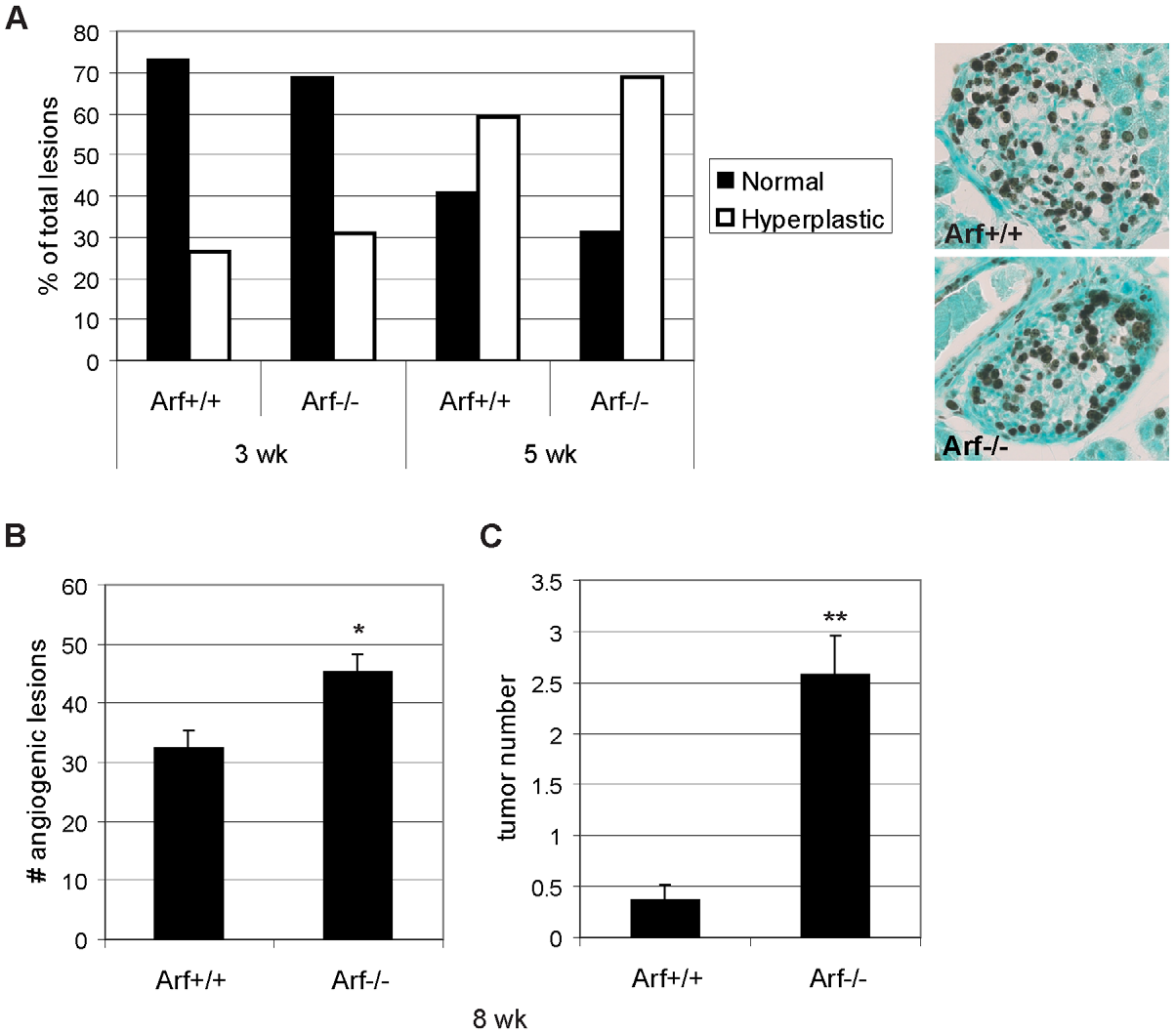
Effects of *Arf* knockout on the hyperplastic and angiogenic switches during RIP-Tag2 tumorigenesis. (A) Effects on the hyperplastic switch. Islets from Ki67-stained sections from 3- or 5-week old mice were scored as “normal” (no hyperproliferation) or hyperplastic. Values represent the distribution of the different islet subclasses within the indicated genotypes. For 3-week mice, 60 (*Arf+/+*) and 58 (*Arf−/−*) islet lesions were scored on sections from 3 mice/group; for 5-week old mice, 166 (*Arf+/+*) and 231 (*Arf−/−*) islet lesions were scored on sections from 3–4 mice/group. The distribution of islet types was not significantly different between the two genotypes at either time point (Fisher's exact test; 5 week timepoint: p = 0.06). Representative Ki67-stained hyperplastic lesions (from 3-week old RIP-Tag2 mice) of the indicated *Arf* genotypes are depicted on the right. (B, C) Effects on the angiogenic switch and tumor initiation. The number of red, angiogenic lesions (B) and tumors (C) was calculated from 11 (*Arf+/+*) or 15 (*Arf−/−*) 8-week old RIP-Tag2 mice of the indicated genotypes. Mean values ±SEM are indicated. Mann-Whitney test for statistical significance; *p = 0.01, **p<0.0001.

To investigate how knockout of *Arf* might be accelerating tumor growth without alterations in β-cell proliferation or apoptosis, we assessed whether loss of Arf could be affecting any of the inherent barriers that limit transition of oncogene expressing β-cells in pancreatic islets through the different steps to tumor formation. Despite expression of the Tag oncogene in the developing pancreas beginning at embryonic day 9, there is a delay in the sporadic initiation of islet hyperproliferation by several weeks. To determine whether loss of Arf could facilitate an earlier initiation of the hyperplastic switch, we examined the percentage of hyperproliferative islet lesions (as determined by staining with the Ki67 proliferation marker) at both 3- and 5-weeks of age in *RIP-Tag2; Arf^+/+^* and *Arf^−/−^* mice. No significant difference in the percentage of hyperproliferative islets was observed at either of these stages (Figure 4A), though at 5-weeks of age, islets from *RIP-Tag2*; *Arf^−/−^* mice were on average larger in size (Figure S3A; 1.5-fold increase; p = 0.007). The increased lesion size was not a result of increased cell size as the number of cells/area was nearly identical between the two groups (Figure S3B).

We next assessed the effects of *Arf* knockout on the frequency of angiogenic switching. In contrast to the negligible impact on the incidence of islet hyperplasias, the number of red, hemorrhagic angiogenic islets was significantly increased (by approximately 30%) in 8 week-old *RIP-Tag2*; *Arf^−/−^* mice compared to *RIP-Tag2*; *Arf^+/+^* littermates (Figure 4B; p = 0.01). In addition, while the appearance of solid tumors was very rare at 8 weeks in *RIP-Tag2*; *Arf^+/+^* mice (tumors present in 4/11 mice; average of <1 tumor/mouse), small tumors were found in nearly all *RIP-Tag2*; *Arf^−/−^* mice analyzed at this age (average of ∼2.5 tumors/mouse; Figure 4C, p<0.0001). Of note, in the scoring for hyperplastic lesions at the 5 week time point, occasional islets exhibiting the typical hemorrhagic features/blood islands of angiogenic islets [22], [23] were observed, with a trend towards a higher incidence in the *Arf^−/−^* compared to *Arf^+/+^* mice; however, this difference was not statistically significant at this time point (Figure S3C). Thus, in this multi-stage model, loss of Arf accelerates tumor formation, at least in part, via facilitating the angiogenic switch.

### Arf knockout does not result in significant alterations to the vascular phenotype

**Figure 5:**
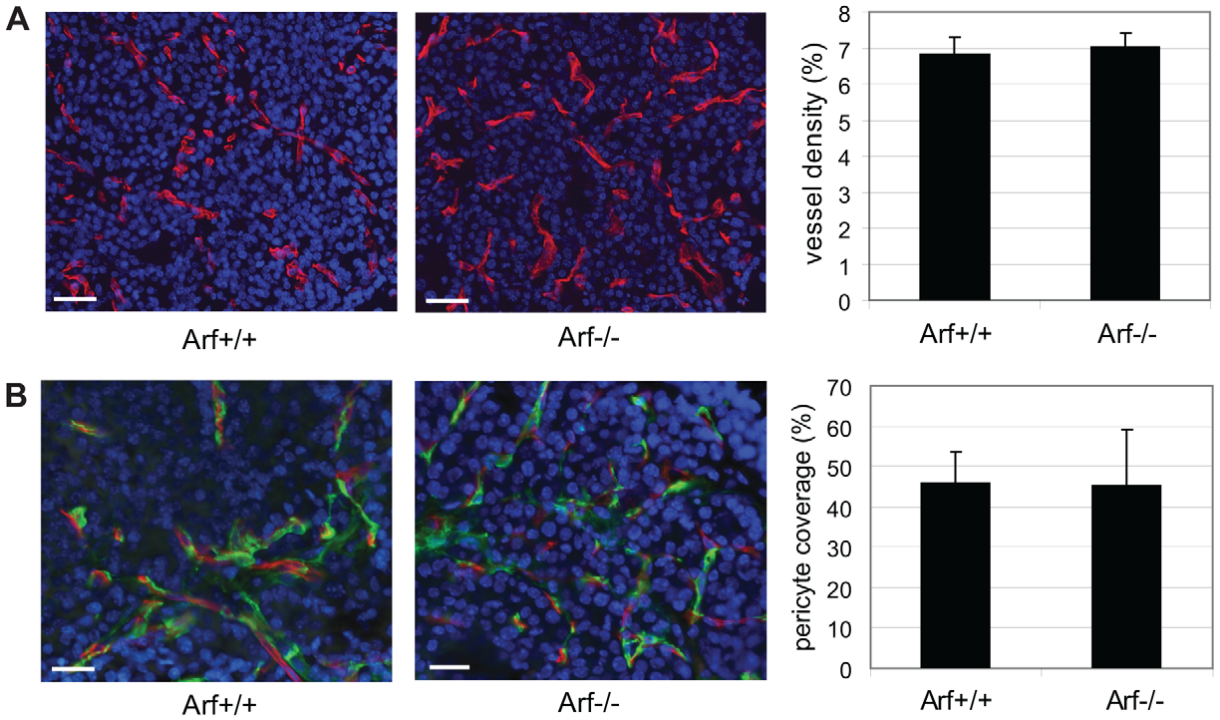
Effects of *Arf* knockout on the angiogenic vasculature. (A) Visualization of the vasculature in angiogenic lesions from 8-week old RIP-Tag2 mice of the indicated genotypes by immunostaining for Meca32 (red). Representative Meca32/DAPI (blue) merged images are shown. Vessel density was calculated by measuring the area occupied by Meca32+ vessels; values represent the mean ±SEM calculated from analyzing 16–20 angiogenic lesions from 4 individual mice of each genotype. (B) Double immunostaining for Meca32 (endothelial cells, red) and NG2 (pericytes, green) was performed to analyze the degree of pericyte coverage within the angiogenic vasculature from 8-week RIP-Tag2 mice of the indicated genotypes. Bars, 20 µm.

To further explore the nature of the increased angiogenic phenotype in the *RIP-Tag2*; *Arf^−/−^* mice, we next assessed the phenotype of the angiogenic vasculature. Despite the increased number of angiogenic lesions in *RIP-Tag2*; *Arf^−/−^* mice, the resultant lesions did not significantly differ in vascular density or morphology (vessel thickness, degree of branching) (Figure 5A and data not shown), or in the degree of pericyte coverage (Figure 5B). Given previous findings demonstrating a role for MMP-9 expressing neutrophils in promoting the angiogenic switch [24], we also examined whether loss of Arf expression resulted in alterations in the incidence of neutrophil recruitment to “pre-angiogenic” hyperplastic lesions. In both *RIP-Tag2*; *Arf^+/+^* and *RIP-Tag2*; *Arf^−/−^* mice, double-immunostaining for the Ki67 proliferation marker and the 7/4 neutrophil marker demonstrated that approximately 25% of hyperplastic lesions examined (at 5 weeks of age) contained infiltrating neutrophils (in most cases not more than one 7/4+ cell/islet; data not shown). In addition, no significant changes in the number of infiltrating MMP-9 and 7/4 double positive cells was observed in angiogenic lesions from 8-wk old *RIP-Tag2*; *Arf^+/+^* and *RIP-Tag2*; *Arf^−/−^* mice (data not shown).

### Selective *Arf* knockout in stromal compartment does not significantly enhance tumor development

**Figure 6:**
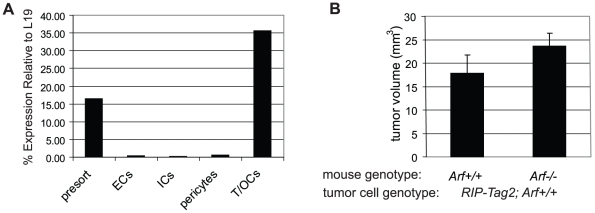
*Arf* expression and the effect of knockout in tumor stromal compartment. (A) Expression of *Arf* in the different constituent RIP-Tag2 tumor cell types. Real-time quantitative RT-PCR was performed on mRNA isolated from FACS sorted cells from RIP-Tag2 tumors [ECs (endothelial cells), ICs (immune cells), T/OCs (tumor/other cells)]. Expression levels plotted relative to L19 expression. (B) Effect of *Arf* knockout on tumor development of orthotopically transplanted tumor cells. 1×10^5^ tumor cells derived from a *RIP-Tag2*; *Arf^+/+^* tumor were injected into the pancreas of *Arf^+/+^* or *Arf^−/−^* mice and allowed to grow for 4 weeks. Values represent the average tumor volume (+SEM) calculated from tumors isolated from 12 individual mice of each of the indicated genotypes. The difference between the values did not achieve statistical significance (Mann-Whitney test; p = 0.1).

Previous studies have demonstrated that *Arf^−/−^* mice are blind due to a defect in involution of the hyaloid vasculature in the eye during development, resulting from an accumulation of Pdgrfrβ+ perivascular cells [25], [26]. Data implicated both cell autonomous and non-autonomous effects of loss of Arf on this phenotype [27]. Since *RIP-Tag2*; *Arf^−/−^* mice lack Arf expression throughout the body and not just in tumor cells, we questioned whether loss of Arf in a non-β-cell type could in part be facilitating the angiogenic switch and tumor formation. To begin exploring this possibility, we first examined whether we could detect *Arf* expression in the stromal compartment of wild-type *RIP-Tag2* tumors. Real-time quantitative RT-PCR analysis on sorted cell compartments from *RIP-Tag2* tumors demonstrated *Arf* expression, as expected, in the tumor cell compartment, but not in endothelial cells (CD31+), immune cells (CD45+), or pericytes (Pdgfrβ+) (Figure 6A and S4). These results are consistent with the notion that induction of Arf expression is, for the most part, limited to response to oncogenic signals [4]; however, this analysis did not rule out the possibility of transient induction of Arf expression in the stromal cell types analyzed, nor expression in other classes of tumor infiltrating cells [e.g. Pdgfrβ-negative pericytes [28]].

To directly assess the potential role of *Arf* knockout in the non-tumor cell compartment in influencing tumor progression, we performed orthotopic experiments wherein tumor cells derived from a *RIP-Tag2*; *Arf^+/+^* tumor were injected into the pancreas of immunocompetent *Arf^+/+^* or *Arf^−/−^* mice, and tumor size was measured four weeks later. There was no significant difference in the average tumor size between tumors formed in *Arf^+/+^* versus *Arf^−/−^* mice (Figure 6B). Combined with our inability to detect *Arf* expression in a tumor stromal cell type, these results suggest that knockout of *Arf* in the tumor stromal compartment is not likely to be the predominant driver of the hastened tumor growth observed upon *Arf* knockout in RIP-Tag2 mice. Since it is unclear whether the mechanism of angiogenesis induction in orthotopically injected tumor cells follows a similar pathway as angiogenesis induced in endogenous pre-neoplastic lesions, the possibility remains that the lack of observed effect could in part be reflecting such differences.

### Knockout of *Arf* accelerates tumor formation via both p53-dependent and independent mechanisms

**Figure 7:**
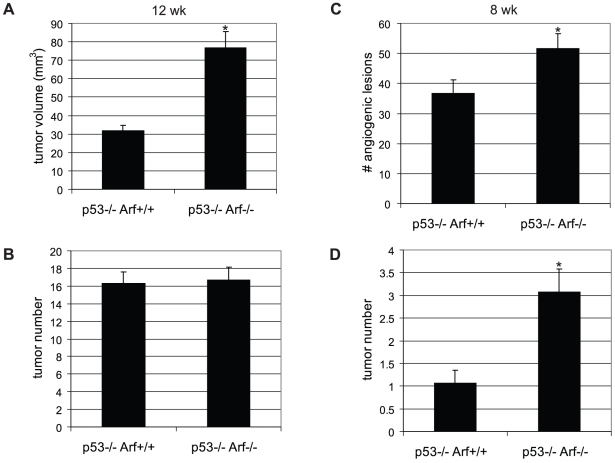
*Arf* knockout accelerates tumor progression in part via p53-independent mechanisms. (A, B) *Arf* knockout in *RIP-Tag2; p53^−/−^* mice results in an increased tumor burden (A; p<0.0001) without affecting tumor number (B) at 12 weeks of age. Mean values +/−SEM from 19 (*p53−/−; Arf+/+*), or 14 (*p53−/−; Arf−/−*) mice/group are indicated. Mann-Whitney test for statistical significance. (C, D) Increased angiogenic islet numbers (C; p = 0.04) and tumors (D; p = 0.002) in *RIP-Tag2; p53^−/−^*; *p19^Arf−/−^* mice at 8 weeks of age. Mean values +/−SEM from 15 (*p53−/−; Arf+/+*), or 13 (*p53−/−; Arf−/−*) mice/group are indicated. Mann-Whitney test for statistical significance.

Since p53 has been shown to be quantitatively sequestered by SV-40 Tag and its transactivation activity is demonstrably inhibited in β-cells of RIP-Tag2 mice [17], we suspected the majority of effects observed upon knockout of *Arf* to be p53-independent. Nevertheless, we proceeded to verify the p53-independence of the *Arf* knockout phenotype by analyzing the effects of *Arf* knockout in a p53-null genetic background. As in the p53-wild-type background, *Arf* knockout in *RIP-Tag2; p53^−/−^* mice resulted in a significantly increased (2.4-fold) tumor burden at 12 weeks of age compared to *Arf^+/+^* littermates (Figure 7A; p<0.001), as well as a significant 30% increase in the number of angiogenic lesions (Figure 7C; p = 0.04) and small tumors (Figure 7D; p = 0.002) at 8 weeks of age, confirming that these effects are at least in part p53-independent. However, it should be noted that the magnitude of the impact of *Arf* knockout on tumor burden (at 12 weeks) and tumor number (at 8 weeks) was not as dramatic as in the *RIP-Tag2*; *p53*-wild-type mice (Figure 2). In addition, although tumor initiation occurred earlier in *RIP-Tag2; p53^−/−^; Arf^−/−^* compared to *RIP-Tag2; p53^−/−^; Arf^+/+^* mice, by 12 weeks of age, no difference in tumor number was detected between mice of these genotypes (Figure 7B), suggesting that both p53-dependent and independent functions of Arf cooperate to suppress the angiogenic switch and tumor progression in this multi-stage carcinogenesis model.

In an attempt to identify a molecular mechanism for the increased angiogenic switching frequency in *Arf-*null RIP-Tag2 mice, we analyzed expression of an array of angiogenesis-related genes by real-time PCR. We did not, however, identify any angiogenic regulatory genes that were convincingly altered at the transcriptional level in whole tumors or angiogenic islets upon loss of Arf (data not shown and Figure S5A). In light of recent studies demonstrating a role for Arf in ribosome biogenesis [29] and in post-transcriptional control of VEGF expression [30], we assessed possible changes in VEGF expression at the protein level upon loss of Arf. Although VEGF protein levels were modestly elevated in RIP-Tag2 tumors in the *p53*-null genetic background (35% increase; p = 0.02), we found that in this genetic context, loss of *Arf* did not result in altered levels of total VEGF protein (Figure S5B). In addition, immunostaining for VEGF did not reveal any obvious changes in VEGF expression/localization in the different pre-neoplastic islet lesions in the *Arf*-null RIP-Tag2 mice (Figure S5C). Thus, there is no indication that the accelerated angiogenesis resulting from loss of Arf involves alteration in VEGF biosynthesis.

## Discussion

Inactivation of the ARF/p53 pathway is a critical event in the majority of human cancers, serving to lift restraints on oncogenic signaling [31]. In addition to the canonical tumor suppressor activities of p53 in mediating cell cycle arrest, apoptosis and senescence, p53 can also function in maintaining tumor dormancy via suppression of angiogenesis [32]. In a multi-stage carcinogenesis model in which p53 is functionally inhibited by SV-40 Tag, we demonstrate that p19^Arf^ can also modulate the angiogenesis program, at least in part via p53-independent mechanisms. Genetic ablation of *Arf in* RIP-Tag2 mice resulted in a significant acceleration of the tumorigenesis pathway, which was associated with an increased frequency of islets undergoing an angiogenic switch and earlier tumor formation. Notably, the hyperproliferative switch was not affected, nor were proliferation/apoptosis indices at any stage of the pathway to tumor formation.

Despite the increased frequency of angiogenic switching, the vasculature within the resultant angiogenic lesions did not significantly differ phenotypically between *RIP-Tag2; Arf^+/+^* and *Arf^−/−^* mice, consistent with previous studies in this model wherein altered angiogenic switching frequencies occurred without concomitant changes in vessel morphology or tumor cell apoptosis/proliferation upon genetic knockout of MMP-9 [33] or neutrophil ablation [24]. The absence of global differences in the degree of neutrophil recruitment to pre-angiogenic lesions in *RIP-Tag2; Arf^−/−^* compared to *Arf^+/+^* mice suggests that Arf may modulate angiogenesis via an alternative mechanism. However, due to the multi-focal and stochastic nature of this tumor model, in which only approximately 15–20% (based on a total of 400 islets/mouse) of hyperplastic lesions will become “angiogenic” (this frequency is increased by just a few % in *Arf^−/−^* mice) the possibility remains that undetected transient alterations in neutrophils in a small subset of lesions could be accounting for the increased incidence of angiogenic switching.

While it is currently unclear what specific signal(s) drives the initial recruitment of inflammatory cells and initiation of the angiogenic switch, the current paradigm suggests that a disruption in the balance between endogenous pro- and anti-angiogenic factors is critical [34]. Notably, of the many p53-independent functions of ARF that have been described to date [8], several can be envisioned to potentially impact the angiogenic switch. In particular, NF-κB and c-Myc, both of which have been implicated in regulating angiogenesis via their effects on expression of pro-inflammatory mediators [35], [36], [37], have been identified as p53-independent targets of ARF [38], [39]. Additionally, nucleolar sequestration of the major inducer of angiogenesis, HIF-1α by ARF has been demonstrated to regulate its transcriptional activity [40]. Further, a recent complementary study by Kawagishi et al. suggested a p53-independent role for ARF in suppressing tumor angiogenesis via post-transcriptional control of VEGF-A expression [30]; however, in the RIP-Tag2 PNET tumors, VEGF protein levels were not significantly altered by loss of Arf. Thus, we infer that increased expression levels of VEGF do not underlay the earlier angiogenic switching, although we cannot exclude possible alterations in VEGF bioavailability, mediated for example by increased matrix degrading proteolytic activity.

A potential role for ARF in the process of vasculogenesis and/or angiogenesis had previously been postulated [8], stemming from the demonstration that Arf can regulate vascular development in the mouse eye [25], one of the only described functions of ARF aside from tumor suppression. A mechanism whereby loss of Arf enhances Pdgf signaling, resulting in an excessive proliferation and accumulation of Pdgfrβ+ perivascular cells was proposed to explain the vascular defect resulting in blindness in *Arf^−/−^* mice. While there were no measurable alterations in pericyte accumulation in the vasculature of *RIP-Tag2; Arf^−/−^* angiogenic lesions, the possibility remains that the initial recruitment of Pdgrβ+ perivascular cells to the neovasculature could be stimulated either directly or indirectly (e.g. via secretion of an as yet to be identified pro-angiogenic factor from Arf-null pre-neoplastic/tumor cells) by loss of Arf.

Expansive tumor development/growth requires not only deregulation of cell growth and survival signals, but also involves significant alterations to the incipient tumor microenvironment. In particular, the formation of a blood supply is critical for tumor growth and metastasis. Emerging evidence suggests that oncogenes and tumor suppressors can influence tumor development not only via cell-autonomous functions in regulating proliferation/apoptosis but also via direct modulation of angiogenesis regulators. The Myc oncogene has been shown to regulate angiogenesis via direct induction of the pro-inflammatory mediator, IL-1β, which promotes MMP activation and release of sequestered VEGF from the extracellular matrix [41]. Ras, via activation of Myc, can activate angiogenesis via repression of the angiogenesis inhibitor, thrombospondin-1 [42]. Similarly, tumor suppressor p53 has been shown to positively regulate thrombospondin-1 expression [43], [44], as well as the production of the anti-angiogenic collagen-derived fragments endostatin and tumstatin [45]. We herein uncover a role for p19^Arf^ in limiting the tumor angiogenic switch, demonstrating that loss of Arf expression promotes the angiogenic switch and hastens tumor development, via both p53-dependent and independent mechanisms, in a model of multi-stage carcinogenesis. These findings lend support to the mounting evidence that Arf can, in part, function as a tumor suppressor independently of its role in modulating p53 activity, and support the growing paradigm that oncogenes/tumor suppressors regulate tumor progression not only via disrupting the balance between homeostatic proliferation/apoptosis but also via eliciting adaptation of the tumor microenvironment to support neoplastic growth.

## Materials and Methods

### Ethics Statement

All animals were handled in strict compliance with the requirements of the Animal Welfare Act and Regulations, the National Institute of Health Guide for the Care and Use of Laboratory Animals, the Public Health Service Policy on the Humane Care and Use of Laboratory Animals, and University of California, San Francisco (UCSF) Policies and Guidelines; mouse experiments were approved by the UCSF Institutional Animal Care and Use Committee (IACUC) (Protocol #: AN079620-03A).

### Transgenic mice breeding

The generation and characterization of *RIP-Tag2* transgenic mice [13] and *p19^Arf^* knockout mice [1] have been previously described. *p19^Arf−/−^* mice were backcrossed 8 generations into C57BL/6 and intercrossed with *RIP-Tag2* mice (C57BL/6) to generate *RIP-Tag2; Arf^+/+^*, *Arf^+/−^*, and *Arf^−/−^* mice. For analyses of the p53-independence of the *RIP-Tag2; Arf^−/−^* phenotype, mice were intercrossed with *Trp53^−/−^* mice [46] in the C57BL/6 background. Littermate controls were used in all experiments.

### Tissue preparation

Normal (from non-transgenic C57Bl/6 mice), hyperplastic islets (from 5–6 wk *RIP-Tag2* mice; not red), and angiogenic islets (from 8–10 wk *RIP1-Tag2* mice; red, hemorrhagic) were isolated as previously described [22]. Tumors were microdissected from the exocrine pancreas of 13–14 wk old RIP1-Tag2 mice. For Taqman analysis, RNA was isolated using the RNeasy Mini kit including the on-column DNase digestion step (Qiagen, Valencia, CA) and cDNA prepared with iScript (BioRad, Hercules, CA). For histology/immunostaining experiments, mice were anesthetized with 2.5% avertin and heart perfused with PBS followed by formalin. Samples were post-fixed for 2 hrs in formalin, followed by infiltration with 30% sucrose prior to freezing in O.C.T. medium; for paraffin embedding, tissue was post-fixed overnight at 4°C in zinc-buffered formalin and processed as previously described [47].

### Real-time RT-PCR gene expression analysis

Real-time RT-PCR was performed by the UCSF Cancer Center Genome Analysis Core on an ABI 7900 machine. For quantitative real-time PCR analysis, gene expression was examined using 3–5 ng input cDNA and all reactions were performed in triplicate; relative expression levels were determined using *L19*, *Gus* and either *Gapdh* or *Cyclophilin* as control genes. Quantitative RT-PCR was performed using the following Taqman assays [all commercially available from Applied Biosystems (Foster City, CA) except for *p19-Arf* (from IDT, Coralville, IA)]: p19-Arf: forward primer: 5′-AGA GGA TCT TGA GAA GAG GGC C-3′, reverse primer: GCA GTT CGA ATC TGC ACC G, probe: 5′-/56-FAM/AAT CCT GGA CCA GGT GAT GAT GAT GGG/36-TAMSp/-3′; PDGFR-β: Mm00435546_m1; Pecam1/CD31: Mm00476702_m1 Ptprt/CD45: Mm00448463_m1, Col1a1: Mm00468761_m1, Vegfa: Mm01281447_m1.

### Analysis of RIP-Tag2 tumor burden and pre-tumor stages

For tumor burden and tumor number analysis, tumors (lesions ≥1 mm) were microdissected and dimensions measured with a ruler. Volumes were calculated using the formula for approximating the volume of a spheroid (volume  =  width^2^ × length ×0.52) and tumor burden calculated as the sum of the volumes of all tumors per mouse. Statistical significance was determined using the Mann-Whitney non-parametric t-test. For angiogenic islet counting, pancreata from 8 week-old mice were dissected, cut into small pieces, and red, hemmorhagic islets (as described in [22]) were scored underneath a dissecting scope. Hyperplastic islets were scored by staining paraffin tissue sections from 3 or 5 week-old mice with a rabbit polyclonal antibody to the Ki67 proliferation marker (clone SP6; Thermo Fisher Scientific, Fremont, CA). Since due to ongoing islet maturation there is a significant degree of β-cell proliferation in islets from non-transgenic mice at 3 weeks of age (compared to minimal β-cell proliferation at 5 weeks of age), to distinguish physiological proliferation from SV40 Tag-induced hyperproliferation, the average percentage of Ki67+ cells in 3-week non-transgenic islets as well as the average percentage from obviously hyperplastic/dysplastic islets in 3-week *RIP-*Tag2 mice was determined and a cutoff of >30% Ki67+ cells was made to define hyperplastic islets. Angiogenic islets were scored histologically by the presence of blood islands/lakes [23] (emptied of blood cells as a result of vascular perfusion method).

### Apoptosis and proliferation assays

TUNEL and BrdU assays were performed on 5 µm paraffin sections as previously described [47]. The percentage of cells in M-phase was determined by immunostaining paraffin sections with a rabbit anti-phospho-Histone H3 antibody (1∶1000, Upstate #06-570, Lake Placid, NY) following antigen retrieval with Citra solution (BioGenex, San Ramon, CA). For assessment of the effects of Arf knockdown on growth of β-tumor cells (βTC) in vitro, the pGIPZ lentiviral shRNAmir delivery system (Thermo Scientific-Open Biosystems, Huntsville, AL) was used to generate cells containing a stable knockdown of Arf. A βTC line derived from a *RIP-Tag2; Arf^+/+^* tumor (βTC105.2) was infected with lentivirus expressing the mouse GIPZ shRNAmir clone V3LHS_646508 for Arf knockdown or a non-targeting shRNAmir control (according to manufacturer's instructions). Infected cells were selected and maintained with 2 µg/ml puromycin. Knockdown of Arf protein expression was confirmed by disrupting cells in RIPA buffer containing a protease inhibitor cocktail (Roche) and immunoblotting equal protein amounts with an antibody to19^Arf^ (5-C3, Abcam). 250,000 cells were plated in a 6-well plate in triplicate and cells were trypsinized and counted on day 2, 4, and 7 to monitor growth.

### Analysis of vasculature

Fixed frozen 10 µm pancreas sections from 8-week old mice were immunostained with a rat monoclonal anti-MECA-32 antibody (1∶200; BD PharMingen, San Diego, CA) to label vessel endothelial cells in the presence or absence of a rabbit polyclonal antibody to the NG2 pericyte marker (1∶200; Chemicon/Millipore). Staining was visualized with FITC or Rhodamine Red-X conjugated secondary antibodies (Jackson ImmunoResearch, West Grove, PA). Vessel density and percentage pericyte coverage of vessels (pixel overlap between MECA-32 and NG2 staining) were determined using MetaMorph software (angiogenesis application) on photographs of representative 200× fields of angiogenic islets.

### Flow cytometry

Tumors were excised from ∼14-week old RIP-Tag2 mice and the major constituent cell types were sorted by FACS as previously described [48] using the following antibodies to sort out endothelial cells, inflammatory cells, and pericytes respectively: CD31-FITC (BD Pharmingen), CD45-APC (BD Pharmingen), and PDGFRβ-PE (eBioscience, San Diego, CA). Cells were sorted into RLT buffer (Qiagen) containing β-ME and stored at −80°C until isolation of RNA using the RNeasy Micro Kit (Qiagen).

### Orthotopic transplant experiment

Pancreata of either *Arf^+/+^* or *Arf^−/−^* mice were injected orthotopically with 100,000 *Arf^+/+^* β-tumor cells (βTC105.2) in a 50 µl volume containing a 1∶1 mixture of cells resuspended in PBS and Growth Factor Reduced Matrigel (#356231, BD Biosciences, Franklin Lakes, NJ). Tumors were excised and measured 4 weeks post-injection.

### VEGF ELISA

VEGF-A concentration was measured using the Quantikine Mouse VEGF Immunoassay (R&D Systems, Minneapolis, MN). Tumor extracts containing 5 µg of total protein were analyzed/well in duplicate following manufacturer's instructions. VEGF concentration was determined using SoftMax Pro software.

### VEGF Immunohistochemistry

5 µm paraffin sections were stained with a goat polyclonal antibody that recognizes the mouse 120 and 164 VEGF-A isoforms (R&D Systems) following antigen retrieval with Antigen Unmasking Solution (Vector Laboratories). A biotinylated anti-goat secondary antibody (Jackson ImmunoResearch Laboratories) in conjunction with the Vectastain ABC Elite system (Vector Laboratories) and SIGMA *FAST* ^TM^ DAB (3,3′-Diaminobenzidine tetrahydrochloride; Sigma) was used for visualization. Sections were counterstained with hematoxylin and images captured using a 20X objective.

## Supporting Information

Figure S1 Maintenance of wild-type Arf allele in tumors from heterozygous Arf knockout mice. Arf mRNA levels were assessed by quantitative RT-PCR on cDNA generated from individual tumors from *RIP-Tag2*; *Arf^+/+^* or *Arf^−/−^* mice. The average Arf expression levels in tumors from the indicated genotypes are depicted with a yellow bar. Values represent expression relative to the L19 control gene.
(0.20 MB TIF)

Figure S2 Loss of Arf does not alter the percentage of proliferating cells in RIP-Tag2 islet lesions. The percentage of cells in M-phase as determined by phospho-histone H3 staining in hyperplastic and angiogenic islets from RIP-Tag2 mice of the indicated Arf genotypes. Values represent the mean percentage ± SEM from graded lesions of 6 mice/genotype. Arf+/+ (Hyp: n = 14; Ang: n = 18); Arf−/− (Hyp: n = 10; Ang: n = 17).
(0.50 MB EPS)

Figure S3 Phenotype of islet lesions from 5-week old RIP-Tag2; Arf+/+ and Arf−/− mice. (A) Lesion size. Average area/islet lesion depicted from area measurements taken from ≥27 individual islets on frozen sections from 3 mice/group. *p = 0.007 (B) Cell size. The number of cells/islet lesion was counted and the average area/cell calculated. Mean values ± SEM depicted. (C) Left: Islets in Ki67-stained sections were scored as “normal” (no hyperproliferation), hyperplastic, or angiogenic. Values represent the distribution of the different islet subclasses within the indicated genotypes; Arf+/+ (166 lesions scored from 4 mice), Arf−/− (231 lesions scored from 3 mice). The differences in lesion distribution between the two genotypes was not significant (Chi-squared test; p = 0.1). Right: representative H&E stained image of an early angiogenic lesion in a RIP-Tag2; Arf−/− mouse (defined by appearance of blood islands [arrowhead] resulting from hemorrhagic vasculature, here emptied as a result of vascular perfusion technique).
(4.98 MB EPS)

Figure S4 Expression of control genes in RIP-Tag2 sorted cell populations. Real-time quantitative RT-PCR to assess expression of the indicated cell type-specific markers was performed on mRNA isolated from FACS sorted cells from RIP-Tag2 tumors to assess for purity of the sorted fractions. Expression levels of the indicated genes (labeled on top of each graph) plotted relative to L19 expression.
(0.34 MB TIF)

Figure S5 Effect of loss of Arf on VEGF expression. (A) Vegfa mRNA levels were measured by quantitative RT-PCR on cDNA generated from pooled tumor RNA of the indicated genotypes. Each pool consisted of equal amounts of RNA isolated from at least 8 tumors derived from 5–7 mice/group. (B) VEGF-A protein levels as measured by ELISA. Bars represent the average (± SEM) concentration of VEGF in RIP-Tag2 tumors of the indicated genotypes (7–10 individual tumors from 7–8 mice/group were analyzed in duplicate). *p = 0.02 compared to Arf+/+ tumors. (C) Pancreas sections from 8 or 12-week old RIP-Tag2 mice of the indicated genotypes were immunostained with an antibody to VEGF. Representative images (sections from 5 mice/group analyzed) of different classes of islet lesions are depicted (normal: left; hyperplastic: middle; angiogenic: right).
(2.03 MB TIF)
