# Supplementary figures and images for: Loss of p19Arf Facilitates the Angiogenic Switch and Tumor Initiation in a Multi-Stage Cancer Model via p53-Dependent and Independent Mechanisms
Source: PLoS One. 2010 Aug 27;5(8):e12454. doi: 10.1371/journal.pone.0012454 (PMC2929208; doi:10.1371/journal.pone.0012454)

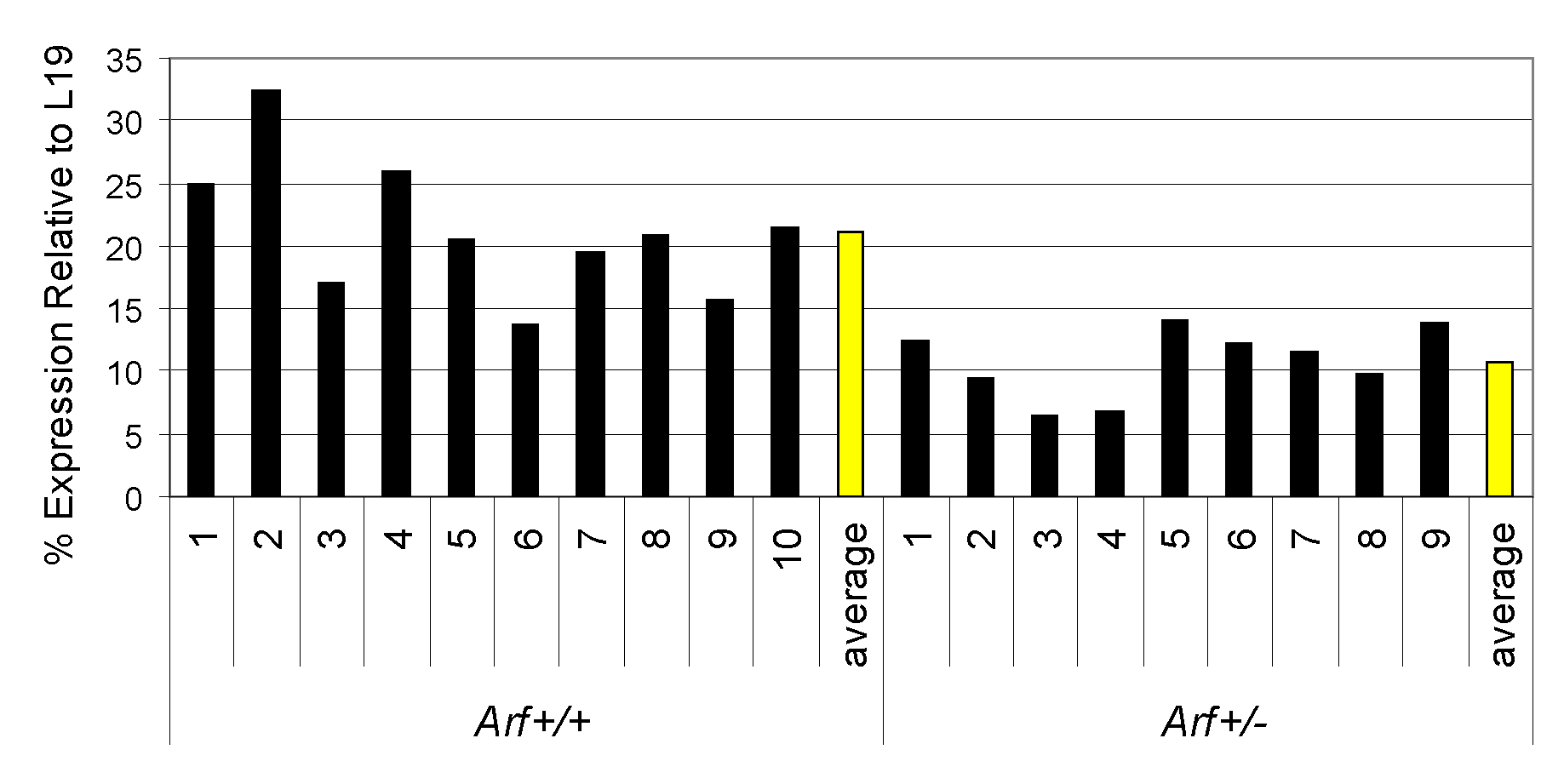

Supplement: Figure S1 — Maintenance of wild-type Arf allele in tumors from heterozygous Arf knockout mice. Arf mRNA levels were assessed by quantitative RT-PCR on cDNA generated from individual tumors from RIP-Tag2; Arf+/+ or Arf−/− mice. The average Arf expression levels in tumors from the indicated genotypes are depicted with a yellow bar. Values represent expression relative to the L19 control gene. (0.20 MB TIF) [file pone.0012454.s001.tif]

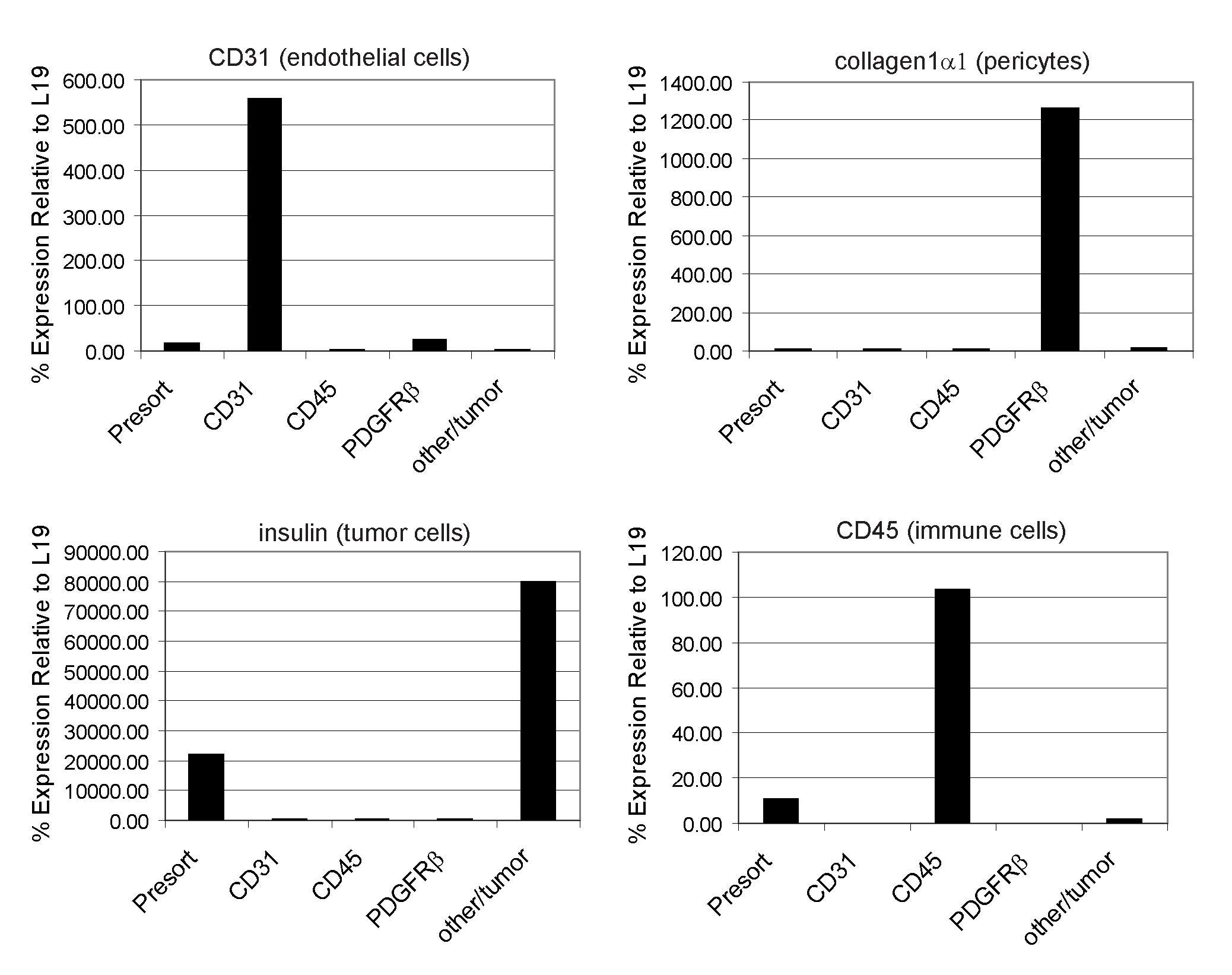

Supplement: Figure S4 — Expression of control genes in RIP-Tag2 sorted cell populations. Real-time quantitative RT-PCR to assess expression of the indicated cell type-specific markers was performed on mRNA isolated from FACS sorted cells from RIP-Tag2 tumors to assess for purity of the sorted fractions. Expression levels of the indicated genes (labeled on top of each graph) plotted relative to L19 expression. (0.34 MB TIF) [file pone.0012454.s004.tif]

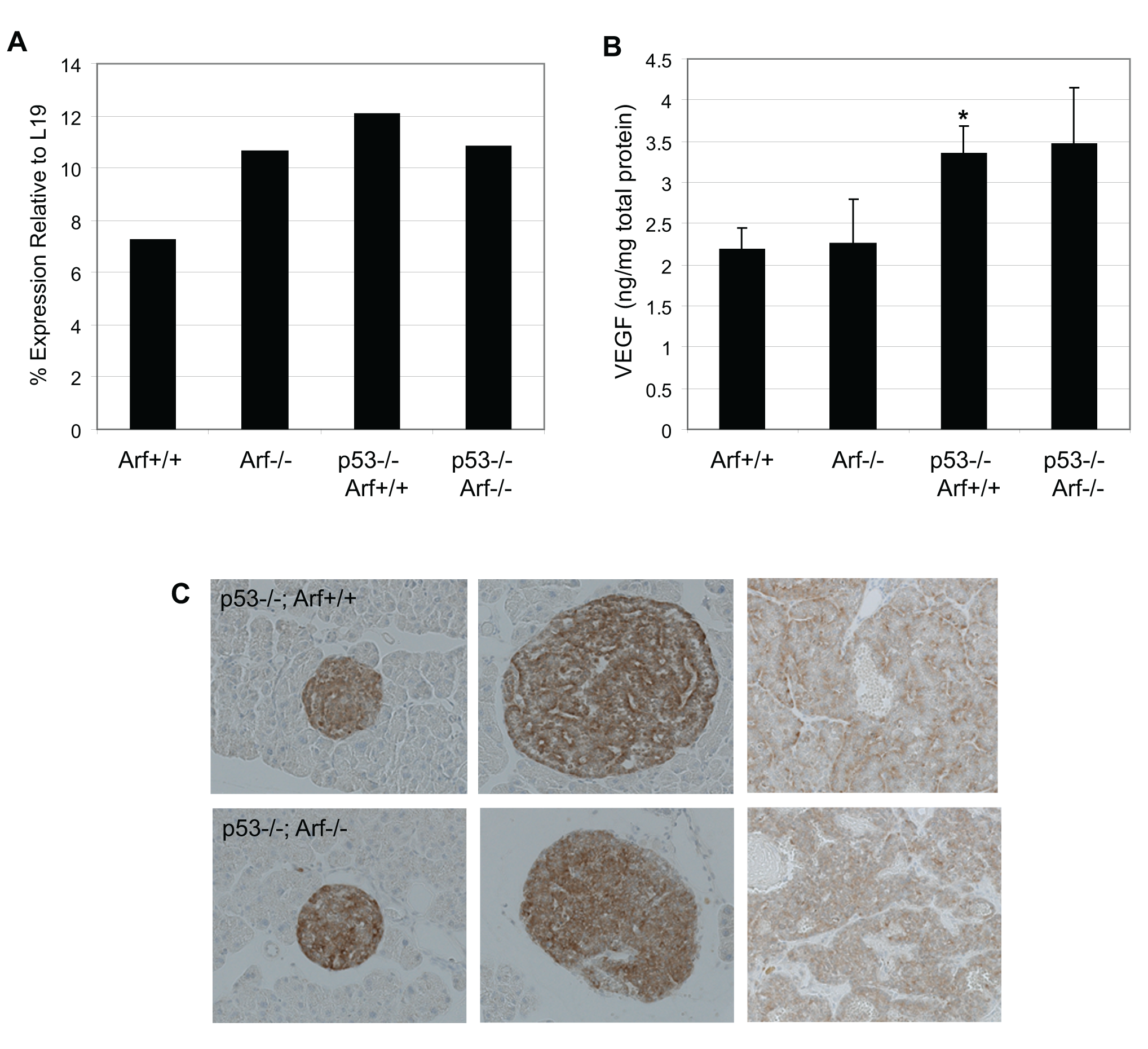

Supplement: Figure S5 — Effect of loss of Arf on VEGF expression. (A) Vegfa mRNA levels were measured by quantitative RT-PCR on cDNA generated from pooled tumor RNA of the indicated genotypes. Each pool consisted of equal amounts of RNA isolated from at least 8 tumors derived from 5–7 mice/group. (B) VEGF-A protein levels as measured by ELISA. Bars represent the average (± SEM) concentration of VEGF in RIP-Tag2 tumors of the indicated genotypes (7–10 individual tumors from 7–8 mice/group were analyzed in duplicate). *p = 0.02 compared to Arf+/+ tumors. (C) Pancreas sections from 8 or 12-week old RIP-Tag2 mice of the indicated genotypes were immunostained with an antibody to VEGF. Representative images (sections from 5 mice/group analyzed) of different classes of islet lesions are depicted (normal: left; hyperplastic: middle; angiogenic: right). (2.03 MB TIF) [file pone.0012454.s005.tif]
